# A Clinico-Epidemiological Study of Neurofibromatosis Type 1 and Its Relation to Quality of Life: A Cross-Sectional Study From India

**DOI:** 10.7759/cureus.22376

**Published:** 2022-02-19

**Authors:** Aditi Mahajan, Milind Patvekar, Samruddhi Lote, Mahendra Singh Deora, Divya Poulose, Jaya Madhurya Gogineni, Krish Panikar, Biswajit Chaklader

**Affiliations:** 1 Dermatology, Dr. D. Y. Patil Medical College, Hospital and Research Centre, Pune, IND; 2 Internal Medicine, A.J. Institute of Medical Sciences, Mangalore, IND; 3 Preventive Medicine, Dr. D. Y. Patil Medical College, Hospital and Research Centre, Pune, IND

**Keywords:** india, dermatology, neurofibromas, dlqi, quality of life (qol), neurofibromatosis 1

## Abstract

Introduction: Neurofibromatosis type 1 (NF1) is an inherited neuroectodermal abnormality with multisystem effects, which can have heavy psychological and physical burdens, especially in countries like India, wherein skin disease is significantly stigmatized. This study was performed to understand the clinical and epidemiological trends of NF1 at a tertiary care center in India and evaluate the association between clinical severity and quality of life in these patients.

Methods: We conducted a cross-sectional study of 40 patients with NF1 over a period of two years at a tertiary hospital in western India. After obtaining consent, demographic and clinical information was collected from the patients and recorded in a pre-designed proforma. Quality of life was assessed by a validated Dermatology Life Quality Index (DLQI) questionnaire in languages understood by the patients and subsequently analyzed.

Results: This study included 40 patients at a mean age of 28.6 years, with a slight male predominance. The most frequently occurring lesions were café-au-lait macules, followed by neurofibromas and intertriginous freckling. The mean DLQI score was 12.35, implying a large effect on most patients' lives. Questions related to self-consciousness, embarrassment, and the influence of skin lesions on clothing choices had the highest scores, indicating a significant effect on social perception.

Conclusion: NF1 has a profound impact on a patient’s quality of life, as evidenced by the high DLQI scores in our patient cohort. The early identification and management of such patients can help prevent further deterioration of their quality of life.

## Introduction

Neurofibromatosis type 1 (NF1) is an inherited neuroectodermal disease, primarily defined by the presence of six or more café‐au-lait macules, intertriginous freckles, two or more neurofibromas, Lisch nodules, bony defects like sphenoid dysplasia, and optic gliomas. It is an autosomal dominant condition with an estimated prevalence of one in 2500-3300 births, with 50% of cases occurring due to sporadic gene mutations [1]. Dermatological manifestations appear early in life and progress with age, but neurological features usually present at a later age [2]. The diagnosis of this condition is usually clinical, with genetic testing rarely being necessary. The cost and accessibility of genetic analysis, which is only available in a few selected tertiary care centers, are major barriers to the research and management of this disease in developing countries like India.

NF1 is a chronic disease that puts a large amount of stress on patients, which adversely impacts their identity, mental well-being, self-confidence, and social relationships. Patients with incurable disorders must cope with multiple impediments to attain a feasible level of health, as well as adequate physical, intellectual, and social functioning [3]. Mental disorders like anxiety, personality disorders, depression, and suicidal tendencies are common in individuals with NF1 [4]. In severe cases, the persistence of cognitive impairment into adulthood hinders a patient's vocational performance, thereby hampering their quality of life (QoL) [5]. In a study of NF1 patients in Iran, Foji et al. found that NF1 affected all aspects of the patient’s life. In particular, perceived social alienation and difficulties regarding marriage and childbirth were present due to apprehensions regarding disease transmission to their progeny [6]. In short, dermatological diseases are a major contributor to the burden of non-fatal disease globally and influence different aspects of an individual's life [7]. Since the initial presentation of NF1 is mainly confined to the skin, patients usually consult dermatologists first. Thus, dermatologists must especially be aware of the options for screening, genetic counseling, and prenatal diagnosis of the disease. A multidisciplinary approach must be adopted for the management of these patients [8-10].

In 1994, Finlay et al. developed the Dermatology Life Quality Index (DLQI) [11,12]. This is a 10-item questionnaire that covers six domains, specifically regarding symptoms and feelings, daily activities, leisure, work and school, personal relationships, and treatment. In this study, we aimed to evaluate the psychological impact of NF1 on an Indian population, gauge their QoL, and understand the extent of disease burden by using the DLQI questionnaire.

## Materials and methods

Study design

This cross-sectional study enrolled 40 patients with NF1 reporting to the dermatology clinic in a tertiary hospital in western India, across a span of 24 months. The study was approved by the Research and Recognition Committee of Dr. D. Y. Patil Medical College, Hospital and Research Centre (approval number: IESC/PGS/2019/43).

Data collection

Socio-demographic and clinical information was obtained from the patients after due consent and documented in a pre-designed proforma. Clinical history and examination of patients were followed by evaluation of their QoL, using a validated DLQI questionnaire (10-item questionnaire) in languages comprehensible to them.

Inclusion and exclusion criteria

All individuals clinically suspected to have NF1 as per the criteria of the National Institute of Health were included in the study. Patients not consenting to participate were excluded from the study. Age, sex, disease duration, and the presence or absence of any other major illnesses were not considered for inclusion or exclusion.

DLQI questionnaire

Each question of the DLQI questionnaire has five possible responses: “not relevant,” “not at all,” “a little,” “a lot,” or “very much,” which are scored as 0, 0, 1, 2, and 3, respectively. Question 7 is scored differently, with “prevented work or studying” being given a score of 3 [13]. The DLQI questionnaire is presented in the Appendix.

The final DLQI score was calculated as the sum of the scores of all questions, which has a range of 0-30. DLQI scores of 0-1, 2-5, 6-10, 11-20, and 21-30, respectively, correspond to having no, small, moderate, very large, and extremely large effect on the patient's life.

Statistical analysis

Data were entered in Microsoft Excel (Microsoft Corporation, Redmond, WA) and analyzed using the Statistical Package for the Social Sciences (SPSS) software version 20 (IBM Corp., Armonk, NY). Categorical variables were expressed in terms of frequency and percentage, whereas continuous variables were expressed as the mean and standard deviation (SD). For qualitative variables, the percentage and chi-square test were performed. Karl Pearson coefficient of correlation was also performed. A two-tailed probability value of 0.05 or less was considered statistically significant.

## Results

This study included 40 patients with an age range of 6-65 (mean: 28.6) years, including 23 (57.5%) males and 17 (42.5%) females (Table 1). A family history of NF1 was positive in 19 (47.5%) patients, with seven (17.5%) patients reporting consanguineous marriage of their parents. Café-au-lait macules and neurofibromas, which were both seen in 38 (95%) patients, were the most commonly occurring lesions. Intertriginous freckling was seen in 28 (70%) patients, Lisch nodules in 19 (47.5%), plexiform neurofibromas in 15 (37.5%), and optic nerve tumors in one (2.5%) patient.

**Table 1:** Demographic factors of NF1 patients. NF1, neurofibromatosis type 1.

Factors	Variants	n	%
Age	10	2	5
	11-20	9	22.5
	21-30	16	40
	31-40	4	10
	41-50	8	20
	>51	1	2.5
Gender	Male	23	57.5
	Female	17	42.5
Family history of NF1	Positive	19	47.5
	Negative	21	52.5
Consanguineous marriage of parents	Present	7	17.5
	Absent	33	82.5

The mean DLQI score was 12.35 ± 7.73, which reflects a significant degree of impact on the QoL of most patients. Table 2 demonstrates the various categories of DLQI scores obtained in our cohort.

**Table 2:** DLQI scores in NF1. DLQI, Dermatology Life Quality Index; NF1, neurofibromatosis type 1.

DLQI categories	n	%
No effect on patient's life	0	0
Small effect on patient's life	10	25
Moderate effect on patient's life	11	27.50
Very large effect on patient's life	10	25
Extremely large effect on patient's life	9	22.50

Table 3 depicts the mean scores of answers to individual questions of the DLQI questionnaire. Impairment of QoL, evaluated via the DLQI questionnaire, revealed that question 2 (i.e., “Over the last week, how embarrassed or self-conscious have you been because of your skin?”) had the highest mean score of 1.9, followed by question 4 (i.e., “Over the last week, how much has your skin influenced the clothes you wear?”), with a score of 1.85. On the other hand, question 6, related to difficulties in playing any sports, had the lowest average score of 0.45.

**Table 3:** Mean scores of individual questions of DLQI. DLQI, Dermatology Life Quality Index.

Question	1	2	3	4	5	6	7	8	9	10	Total
Mean	1	1.9	0.65	1.85	0.95	0.45	1.8	1.43	0.87	1.45	12.35
SD	0.894	0.943	0.823	0.989	1.047	0.705	1.47	0.891	1.053	0.921	7.738

Tables 4, 5 demonstrate the comparison between the various demographic and clinical factors and DLQI categories. Age, presence of café‐au-lait macules, Lisch nodules, freckling, and plexiform neurofibromas had a significant negative correlation with QoL (p < 0.05). Gender, family history of the disease, and parental consanguinity did not have a significant correlation with QoL.

**Table 4:** Comparison between demographic factors and DLQI categories. DLQI, Dermatology Life Quality Index; NF1, neurofibromatosis type 1.

		DLQI categories					P-value
		Small effect	Moderate effect	Very large effect	Extremely large effect	Total	
Gender	Male	7	6	7	3	23	p = 0.35
		30.43%	26.10%	30.43%	13.04%	100.00%	
	Female	3	5	3	6	17	
		17.65%	29.41%	17.65%	35.29%	100.00%	
		DLQI categories					P-value
		Small effect	Moderate effect	Very large effect	Extremely large effect	Total	p = 0.0015
Age	10	2	0	0	0	2	
		18.75%	50.00%	12.50%	18.75%	100.00%	
	31-40	1	0	0	3	4	
		25.00%	0.00%	0.00%	75.00%	100.00%	
	41-50	1	0	4	3	8	
		12.50%	0.00%	50.00%	37.50%	100.00%	
	>51	0	0	1	0	1	
		0.00%	0.00%	100.00%	0.00%	100.00%	
		DLQI categories					P-value
		Small effect	Moderate effect	Very large effect	Extremely large effect	Total	p = 0.17
Family history of NF1	Positive	3	4	5	7	19	
		15.79%	21.05%	26.32%	36.84%	100.00%	
	Negative	7	7	5	2	21	
		33.33%	33.33%	23.81%	9.53%	100.00%	
		DLQI categories					P-value
		Small effect	Moderate effect	Very large effect	Extremely large effect	Total	p = 0.34
Consanguineous marriage of parents	Present	0	2	2	3	7	
		0.00%	28.57%	28.57%	42.86%	100.00%	
	Absent	10	9	8	6	33	
		30.30%	27.28%	24.24%	18.18%	100.00%	

**Table 5:** Comparison between clinical features and DLQI categories. DLQI, Dermatology Life Quality Index; CALMS, café‐au-lait macules; NFs- neurofibromas.

		DLQI categories					P-value
		Small effect	Moderate effect	Very large effect	Extremely large effect	Total	p < 0.0001
CALMS	10	3	2	0	0	5	
		60.00%	40.00%	0.00%	0.00%	100.00%	
	11-25	6	6	1	2	15	
		40.00%	40.00%	6.67%	13.33%	100.00%	
	26-50	0	3	6	4	13	
		0.00%	23.08%	46.15%	30.77%	100.00%	
	>50	0	0	2	3	5	
		0.00%	0.00%	40.00%	60.00%	100.00%	
		DLQI categories					P-value
		Small effect	Moderate effect	Very large effect	Extremely large effect	Total	p < 0.01
NFs	10	6	1	1	0	8	
		75.00%	12.50%	12.50%	0.00%	100.00%	
	11-25	3	8	5	4	20	
		15.00%	40.00%	25.00%	20.00%	100.00%	
	26-50	0	1	2	3	6	
		0.00%	16.67%	33.33%	50.00%	100.00%	
	>50	0	0	2	2	4	
		0.00%	0.00%	50.00%	50.00%	100.00%	
		DLQI categories					P-value
		Small effect	Moderate effect	Very large effect	Extremely large effect	Total	p < 0.001
Freckling	Present in	3	8	8	9	28	
		10.71%	28.57%	28.57%	32.15%	100.00%	
		DLQI categories					P-value
		Small effect	Moderate effect	Very large effect	Extremely large effect	Total	p = 0.007
Lisch nodules	Present in	3	3	7	6	19	
		15.79%	15.79%	36.84%	31.58%	100.00%	
		DLQI categories					P-value
		Small effect	Moderate effect	Very large effect	Extremely large effect	Total	p = 0.006
Plexiform NFs	Present in	0	1	6	8	15	
		0.00%	6.67%	40.00%	53.33%	100.00%	

## Discussion

Our study cohort had a slight male predominance, which was also seen in a study by Purkait et al. on patients with neurocutaneous syndromes [2]. The most frequently occurring lesions in our study were café‐au-lait macules, seen in 95% of patients, which had a similar prevalence of 93% in a study by Lakshmanan et al. on NF1 patients in South India. Furthermore, their study demonstrated freckling in 90% of patients, whereas in our group, this occurred in 70%. A family history of NF1 was positive in 47.5% of our patients, in contrast to 33% of patients in their study [14]. Cutaneous neurofibromas were seen in 95% of our patients, similar to the findings of Lakshmanan et al. and Wolkenstein et al., with an occurrence of 96.6% and 91%, respectively [10,14].

The presence of plexiform neurofibromas in our study was comparable to that of a study by Huson et al. (37.5% vs. 32%, respectively) [15]. On the other hand, Lisch nodules were present in 47.5% of patients in our study, which is lower than that in a study from Northwest England and a study by Friedman et al. (63% and 57%, respectively) [16,17].

The mean DLQI was 12.35 ± 7.73, with higher QoL impairment in females (14 ± 8.66) than in males (11.13 ± 6.72). Higher DLQI scores were observed with more extensive skin involvement, female gender, and the presence of plexiform neurofibromas. Upon analysis of DLQI scores, NF1 had no effect in zero cases but had a small, moderate, very large, and extremely large effect in 10 (25%), 11 (27.5%), 10 (25%), and nine (22.5%) patients, respectively.

Similar studies from Italy and America demonstrated that patients with more visible lesions had a greater impact on the emotional aspect of their QoL [18,19]. A study from Brazil concluded that patients were significantly concerned about transmitting the disease to their progeny and expressed difficulties in obtaining appropriate treatment [20]. Various studies on the QoL of NF1 patients done in a few countries also reported a negative effect on QoL across multiple aspects of life [21,22]. A Canadian study of 176 patients reported that patients' mental well-being and overall QoL were influenced greatly by their perceived physical appearance [23].

The presence of café-au-lait macules, Lisch nodules, plexiform neurofibromas, and freckling had a significant negative correlation with QoL (P < 0.05). Individual questions of the DLQI questionnaire demonstrated the highest mean scores for questions 2 and 4 (1.9 and 1.85, respectively), followed by question 10 (1.8), indicating that self-consciousness, embarrassment, choice of clothing, and time taken by treatment were aspects of their lives most affected by the disease. Questions 3 and 6, pertaining to difficulty in playing sports and shopping/gardening, had the lowest mean scores, signifying a lower impact on these spheres of a patient’s life.

Study limitations

More extensive questionnaires like the Skindex and 36-Item Short Form Survey (SF-36) were not used to evaluate the QoL due to the poor compliance of patients and the relatively lengthy nature of those questionnaires.

## Conclusions

NF1 has a significant effect on patients' QoL due to changes in their appearance and societal stigma. Patients with comparatively fewer lesions can also experience a drastic impairment in their QoL, and their mental health must be considered just as much as a patient with extensive lesions. Early identification of patients with impaired QoL should prompt adequate therapeutic counseling and reference for a multidisciplinary approach.

## Figures and Tables

**Figure 1:**
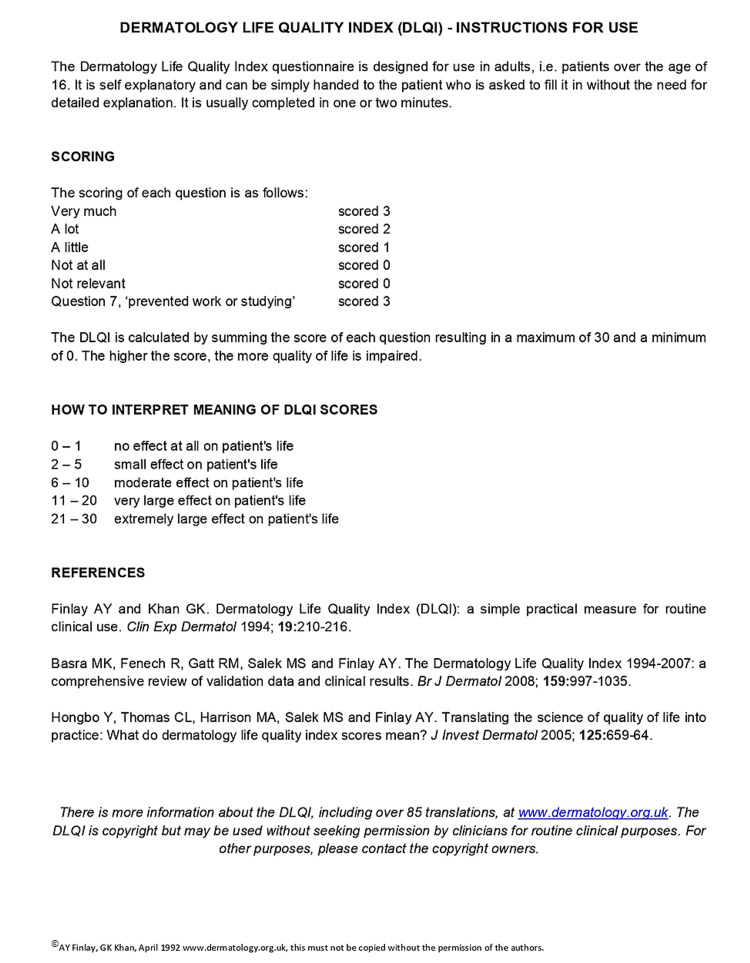
Dermatology Life Quality Index (DLQI) questionnaire (interpretation).

**Figure 2:**
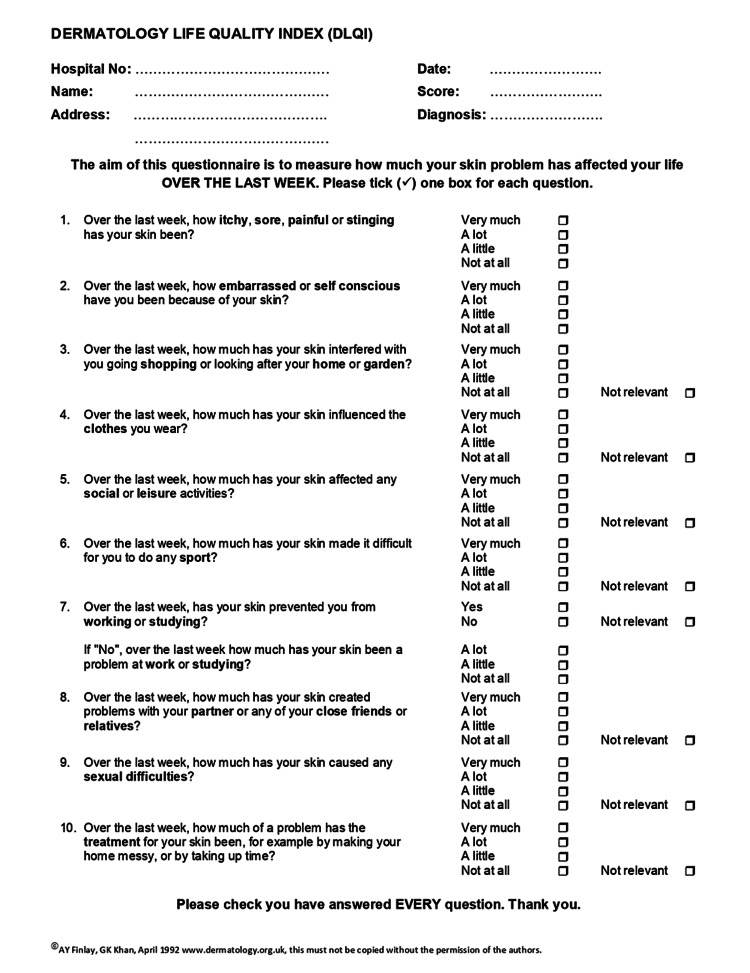
Dermatology Life Quality Index (DLQI) questionnaire.

## Appendices

Dermatology Life Quality Index (DLQI) questionnaire
